# Is It Possible to Improve the Bioavailability of Resveratrol and Polydatin Derived from *Polygoni cuspidati* Radix as a Result of Preparing Electrospun Nanofibers Based on Polyvinylpyrrolidone/Cyclodextrin?

**DOI:** 10.3390/nu14193897

**Published:** 2022-09-21

**Authors:** Magdalena Paczkowska-Walendowska, Andrzej Miklaszewski, Judyta Cielecka-Piontek

**Affiliations:** 1 Department of Pharmacognosy, Poznan University of Medical Sciences, Rokietnicka 3, 60-806 Poznan, Poland; 2 Institute of Materials Science and Engineering, Faculty of Materials Engineering and Technical Physics, Poznan University of Technology, Jana Pawła II 24, 61-138 Poznan, Poland

**Keywords:** *Polygoni cuspidate* extract, nanofibers, resveratrol, polydatin, bioavailability

## Abstract

The low bioavailability of resveratrol and polydatin obtained from *Polygoni cuspidati* extract limits the application of their pro-health properties. While nanofibers have attracted increasing attention in nutrition delivery due to their special properties, including an increase in the dissolution and permeability, which affects the bioavailability. Therefore, it is justified to obtain nanofibers from *Polygoni cuspidati* extract, which showed antioxidant and anti-inflammatory properties as a result of a presence of stilbene analogs in the *Polygoni cuspidati* extract (especially resveratrol and polydatin). In the first stage of the work, using the Design of Experiment (DoE) approach, the *Polygoni cuspidati* extract (70% of methanol, temperature 70 °C and 4 cycles) was obtained, which showed the best antioxidant and anti-inflammatory properties. Using the *Polygoni cuspidati* extract as a substrate, nanofibers were obtained by electrospinning. The identification of nanofibers was confirmed on the basis of the analysis of changes in XRPD diffractograms, SEM picture and FTIR-ATR spectra. Obtaining nanofibers from the *Polygoni cuspidati* extract significantly improved the solubility of resveratrol and polydatin (approx. 6-fold comparing to pure substance). As a consequence, the penetration coefficients of both tested resveratrol and polydatin also increased. The proposed strategy for the preparation of nanofibers from the *Polygoni cuspidati* extract is an innovative approach to better use the synergy of biological action of active compounds present in extracts. It is especially during the development of nutraceuticals based on the use of selected stilbenes.

## 1. Introduction

Epidemiological data indicate that civilization diseases, such as cardiovascular disease, diabetes, overweight, obesity and cancer, are significantly increasing [1]. Key factors important in the development of civilization diseases are a polluted environment, low physical activity and improper diet, as well as an increase in nervous tension and stressful situations. Many of these factors increase the level of free radicals, the presence of which induces the development of many civilization diseases. The reduction of the production of free radicals and their neutralization are the main assumptions of the prevention of the development of many diet-related diseases [2]. Therefore, the formation of ROS is conditioned by ultraviolet radiation, alcohol consumption, smoking and a diet with an insufficient content of compounds with redox properties [3].

Diseases develop if there is no homeostasis in the human body, which is determined by the oxidation–antioxidant balance, and free radicals are not neutralized by antioxidants. Therefore, it is extremely important to take antioxidants, whose task is to maintain the above-mentioned oxidative–antioxidant balance and to prevent the development of civilization diseases. Among the valuable natural antioxidants it is worth pointing out polyphenols, which are secondary specialized plant metabolites [4]. An interesting group of polyphenols is stilbenes, the main representative of which is resveratrol, occurring, inter alia, in wine, grapes and berries. It protects the cardiovascular system by preventing damage to the walls of the arteries. Resveratrol inhibits the growth of cancer cells and protects protein thiol groups against oxidative oxidation. It has antitumor activity by influencing the main stages of carcinogenesis by modulating signaling pathways that control cell division and growth, apoptosis, inflammation, angiogenesis and metastasis [5].

Commercially available resveratrol is often obtained by chemical synthesis. That is why it is so valuable to take plant materials rich in this polyphenol to be able to use the potential of naturally occurring stilbenes. One of the raw materials particularly rich in silbenes is *Polygoni cuspidati rhizoma et radix*. It is worth noting that other secondary metabolites also present in the metabolite pathway of resveratrol formation have valuable pro-health properties. Additionally, *Polygonum cuspidatum,* also known as Hu Zhang in China, has the rich composition of active compounds—epicatechin, resveratrol and polydatin—as well as hydrophobic components, including emodin, physcion, torachrysone and their glycosides [6]. Hu Zhang is also used to treat a wide range of diseases due to its antiviral, antibacterial, anti-inflammatory, neuroprotective and cardioprotective effects [7].

Despite the broad spectrum of health-promoting properties, the active compounds (in particular polydatin and resveratrol) are characterized by low water solubility [8]. Additionally, when administered orally, resveratrol is readily absorbed from the small intestine. Despite rapid systemic absorption, resveratrol achieves low bioavailability, less than 1%, due to its high first-pass metabolism, mainly in enterocytes and the liver [9]. One of the methods of increasing the solubility of active compounds while bypassing enterohepatic metabolism is the production of electrospun fibers with bioactive substances for buccal administration. Solid dispersion is one of the methods that can improve active substances’ dissolution and enhance their bioavailability [10]. One technique that can improve the solubility and, consequently, the bioavailability of active compounds is electrospinning. Due to the large contact surface, the nanofibers formed as a result of this process show the possibility of adhesion to the epithelium of the oral cavity, which makes it possible to bypass the hepatic metabolism in the case of active compounds absorbed within the oral cavity [11]. There are literature data on the production of electrospun nanofibers to improve the properties of polyphenols, and in particular of stilbene derivatives—namely, resveratrol. PVP-based nanofibers improved resveratrol solubility [12,13], while nanoencapsulation using poly (caprolactone) (PCL) has been reported for prolonged release of resveratrol [11] for tissue engineering and wound healing application [14]. Further, electrospun nanofibers with zein protected resveratrol from the adverse pH condition of the stomach and were released at a controlled rate in the intestinal [15]. Interestingly, there are individual reports in the literature on the use of electrospun nanofibers to increase the bioavailability of active substances [16,17]; however, there are no data of electrospun nanofibers’ production with a herbal extract rich with resveratrol.

Therefore, the aim of this study is to develop an innovative preparation of nanofibers containing an extract rich in stilbenes and their associated compounds. Due to the complexity of the extract matrix, this goal was achieved in the next stages: (1) preparation of the extract with the highest contents of compounds (polydatin and resveratrol) as well as biological activity; and (2) preparation of electrospun nanofibers with increased solubility and permeability of active compounds present in the extract with the best antioxidant activity as well as the ability to inhibit hyaluronidase activity.

## 2. Materials and Methods

### 2.1. Plant Material

Plant raw material, *Polygonum cuspidatum* rhizome and root, was purchased from Herbapol Cracow (Cracow, Poland) (Lot No. 010918).

### 2.2. Chemicals and Reagents

Resveratrol (≥99%, HPLC), polydatin (≥95%, HPLC), emodin (phyproof^®^ Reference Substance) and physcion (phyproof^®^ Reference Substance) were obtained from Sigma-Aldrich (Poznan, Poland). Excipients, such as (2-Hydroxypropyl)-β-cyclodextrin (HPβCD) average Mw ~1460, were supplied from Sigma-Aldrich (Poznan, Poland), and polyvinylpyrrolidone (PVP) as Kollidon^®^ 30 was supplied from BASF Pharma (Burgbernheim, Germany). Reagents for activity assays include the following: 2,2-Diphenyl-1-picrylhydrazyl (DPPH), potassium persulfate (K_2_S_2_O_8_), 2,2′-Azino-bis(3-ethylbenzothiazoline-6-sulfonic acid) diammonium salt (ABTS, C_18_H_24_N_6_O_6_S_4_), neocuproine, ammonium acetate, copper(II) chloride (CuCl_2_·H_2_O), sodium acetate trihydrate (CH_3_COONa·3H_2_O), 2,4,6-tris(2-pyridyl)-1,3,5-triazine (TPTZ, C_18_H_12_N_6_), iron(III) chloride hexahydrate (FeCl_3_·6H_2_O), sodium chloride, bovine serum, hexadecyltrimethylammonium bromide (CTAB), hyaluronic acid (HA); for dissolution studies, they include the following: potassium chloride, sodium chloride, di-potassium hydrogen orthophosphate, magnesium chloride, calcium chloride and xylitol; and for mucoadhesive tests, they include the following: mucin from porcine stomach were obtained from Sigma-Aldrich (Poznan, Poland). Prisma™ HT buffer, Acceptor Sink Buffer and GIT lipid solution were obtained from Pion Inc., whereas HPLC grade acetonitrile and water were obtained from Merck. High-quality pure water and ultra-high-quality pure water were prepared using a Direct-Q 3 UV Merck Millipore purification system.

### 2.3. Obtaining and Charakteristion of Biological Activity of Polygoni Cuspidati Extract

#### 2.3.1. Plant Extraction Using Design of Experiment (DoE)

Using the Design of Experiments (DoE) approach, a factor experiment plan was developed for three independent variables, which were assigned three levels of values (3^2^ full factorial design). As independent factors were selected, the content of extraction mixture, its temperature and the number of repetitions of the cycles were identified (Table 1).

The following parameters used to assess extraction efficiency were choosen: sum of content of active components, total content of phenolic compounds and antioxidant (DPPH scavenging assay) as well as antiinflammation activities (inhibition of hyaluronidase activity).

#### 2.3.2. Determination of Selected Active Components Content and Total Phenolic Content (TPC)

The contents of main active compounds (polydatin, resveratrol, emodin and parietin) were determined by using the modified HPLC-Diode-Array Detection method described previously by Paczkowska-Walendowska et al. [8].

The total content of phenolic components was determined by using method described previously [18].

#### 2.3.3. Determination of Biological Activity

##### Antioxidant Activity

Antioxidant activity was determined by using an assay with 2,2-Diphenyl-1-picrylhydrazyl (DPPH), 2,2-Azino-bis(3-ethylbenzothiazoline-6-sulfonic Acid) (ABTS) Radical Cation-Based Assays, Cupric Ion-Reducing Antioxidant Capacity (CUPRAC) Assay and Ferric Ion-Reducing Antioxidant Parameter (FRAP) Assay. All procedures were described previously [18].

##### Anti-Hyaluronidase Activity

The procedure of hyaluronidase inhibition was determined by the turbidimetric method described previously [18].

### 2.4. Obtaining of Electrospun Nanofibers Containing Polygoni Cuspidati Extract

#### Electrospun Nanofibers’ Preparation Using Design of Experiment (DoE)

The electrospinning procedure was performed using NS + NanoSpinner Plus Electrospinning Equipment (Inovenso Ltd., Istanbul, Turkey). The amount of *Polygoni cuspidati* extract, PVP and HPβCD used to prepare nanofibers was selected on the basis of the Design of Experiment (DoE) data and 3^2^ full factorial design experimental plan and is presented in Table 1. The parameters used to assess electrospinning efficiency were as follows: determination of active components content, total amount of released drug, permeability of active compounds and bioadhesion properties of systems.

Firstly, *Polygoni cuspidati* extract (W10—optimized extract prepared under the conditions: 70% of methanol in the extraction mixture, temperature 70 °C and 4 cycles) was mixed with 10 ml of ethanol, and then the appropriate amount of HPβCD was added (Table 2) and stirred with a magnetic stirrer until completely dissolved. Then, the appropriate amount of PVP was added (Table 2) and stirred with a magnetic stirrer until completely dissolved. The evenly mixed solution was transferred to a syringe and subjected to the electrospinning process with the following parameters: voltage of 25 kV, flow rate of 2 mL/min and distance of 12 cm. The nanofibers were collected in a rotary collector covered with aluminum foil. Conditions were optimized based on the preliminary trials.

### 2.5. The Identification of Optimized Electrospun Nanofibers

#### 2.5.1. Scanning Electron Microscopy (SEM)

The surface morphology of the nanofiber was visualized using SEM. The nanofibers were sputter coated with gold-palladium and then visualized by a scanning electron microscope Quanta 250 FEG (FEI, Eindhoven, The Netherlands).

#### 2.5.2. XRPD

The crystallographic structure of the samples was analyzed by an X-ray diffraction (XRD, Panalytical Empyrean, Almelo, Netherlands) equipment with the copper anode (CuKα—1.54 Å) at a Brag-Brentano reflection mode configuration with 45 kV and 40 mA parameters. The measurement parameters were set up for 3–60° with a 45 s per step 0.05° in all cases.

#### 2.5.3. Fourier Transform Infrared Spectroscopy with Attenuated Total Reflectance (FTIR-ATR)

The FTIR-ATR spectra were measured between 400 cm^−1^ and 4000 cm^−1^, with a resolution set to 1 cm^−1^, with a Shimadzu IRTracer-100 spectrometer equipped with a QATR-10 single bounce, diamond extended range and LabSolutions IR software (Warsaw, Poland).

### 2.6. Characterisation of Electrospun Nanofibers

#### 2.6.1. Determination of Active Components Content

Contents of polydatin and resveratrol were determined by using method described in Section 2.3.2.

#### 2.6.2. Dissolution Studies

Dissolution studies of electrospun nanofibers were performed using an Agilent 708-DS dissolution apparatus. A standard basket method was used at 37 ± 0.5 °C with a stirring speed of 50 rpm. Nanofibers were placed in 300 mL of artificial saliva solution at pH 6.8 (potassium chloride (1.20 g), sodium chloride (0.85 g), di-potassium hydrogen orthophosphate (0.35 g), magnesium chloride (0.05 g), calcium chloride (0.20 g), xylitol (20.0 g) and water up to 1 L; pH was adjusted to 6.8 by 1 M HCl). The liquid samples were collected at specified time intervals, and an equal volume of temperature-equilibrated media was replaced. The samples were filtered through a 0.45 μm nylon membrane filter. The concentrations of polydatin and resveratrol in the filtered acceptor solutions were determined by the HPLC method described above. Sink conditions were preserved in the studies.

#### 2.6.3. Permeability Studies

Permeability of active compounds enclosed in nanofibers through artificial biological membranes was investigated by using the PAMPA™ (parallel artificial membrane permeability assay) gastrointestinal tract (GIT) assay (Pion Inc., Billerica, MA, USA). Nanofibers were dissolved in donor solutions (artificial saliva solution at pH 6.8). The acceptor plates were loaded with acceptor Prisma buffer with pH 7.4. The plates were put together and incubated under conditions: temperature 37 °C and 15 min with continuous stirring 50 rpm. Each experiment was repeated at least three times. The amount of permeated active compounds was determined using the HPLC method described above.

The apparent permeability coefficients (P_app_) were calculated from the following equation:

$$P_{app}=\frac{-ln\left(1-\frac{C_{A}}{C_{equilibrium}}\right)}{S\times \left(\frac{1}{V_{D}}+\frac{1}{V_{A}}\right)\times t}$$

where *V*_D_ is the donor volume, *V*_A_ is the acceptor volume, *C*_equilibrium_ is the equilibrium concentration $C_{equilibrium}=\frac{C_{D}\;\times \;V_{D}\;+\;C_{A}\;\times \;V_{A}}{V_{D}\;+\;V_{A}}$, *C*_D_ is the donor concentration, *C*_A_ is the acceptor concentration, *S* is the membrane area and *t* is the incubation time (in seconds).

#### 2.6.4. In Vitro Assessment of Mucin–Biopolymer Bioadhesive Bond Strength

A viscometric method was used to quantify mucin–polymer bioadhesive bond strength. Assessment was performed according to the method described previously [8].

#### 2.6.5. Antioxidant Activity

Antioxidant activity of electrospun nanofibers was conducted by DPPH assay described in Section 2.3.3.

### 2.7. Statistical Analysis

Statistical analysis was carried out with Statistica 13.3 software (TIBCO Software Inc., Palo Alto, CA, USA). The normality of the results was checked using the Shapiro–Wilk test. The differences among the mean values were tested using the ANOVA test with post hoc Tukey’s range test for multiple comparisons. Differences between groups were considered to be significant at *p* < 0.05.

## 3. Results and Discussion

Worldwide interest in using complementary and herbal medicines for the treatment and prevention of various illnesses has grown in recent years. The actual health advantages of diverse herbal products, however, appear to be overshadowed by issues with quality (lack of consistency, safety and efficacy). Extracts from the herbal matrix must be standardized and characterized for use in modern phytopharmaceuticals. The extraction conditions determine the quality and the yield of the individual constituents; therefore, the choice of the extraction method becomes one of the most important stages in the development of modern phytopharmaceuticals [19]. One of the approaches worth implementing for the preparation of herbal extracts is the design of experiment approach, which assesses the influence of input factors (e.g., temperature, the composition of the extraction mixture and extraction method) on the properties of the extract (e.g., content of active compounds and biological activity) [19,20]. In this work, a full factorial design model was created to assess the effectiveness of the extraction process (Table 1).

**Figure 1:**
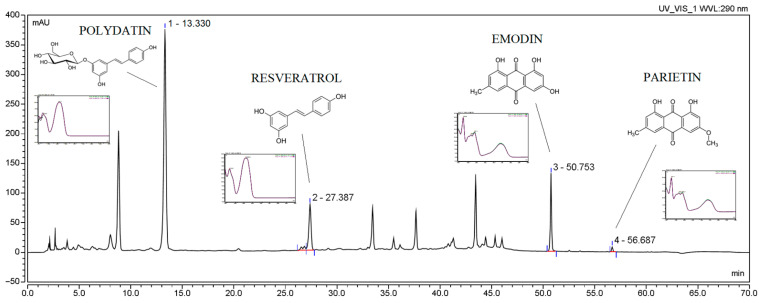
Chromatogram of extract W9.

Firstly, the assessment of active compounds, such as the polydatin, resveratrol, emodin and parietin, was evaluated (Figure 1, Table 3). The HPLC method was validated according to ICH guidelines, and validation parameters are collected in Table S1 (Supplementary Materials).

The content of active compounds is collected in Table 4. All extracts were also tested in regards to the total polyphenol content (Table 4).

Then, the sum of the content of active compounds was analyzed. Based on the Pareto diagram (Figure S1, Supplementary Materials), it can be indicated that the percentage of methanol in the extraction mixture and the temperature are statistically significant factors affecting the content of the sum of active compounds. Both effects have a positive sign, i.e., with the increase in the percentage of methanol in the extraction mixture and with the increase in temperature, the sum of active compounds increases.

The TPC was the one output parameter in the DoE model. Based on the Pareto diagram for the TPC (Figure S2, Supplementary Materials), it can be indicated that the percentage of methanol in the extraction mixture and the temperature are statistically significant factors affecting the content of the TPC. Both effects have a positive sign, i.e., with the increase in the percentage of methanol in the extraction mixture and with the rise in temperature, the TPC increases.

In assessing the impact of extraction parameters on its efficiency, it is crucial to determine the final physical properties and biological activity of the prepared extracts. For this purpose, antioxidant activity (measured by four methods: DPPH, ABTS, CUPRAC and FRAP) and anti-inflammatory activity measured as hyaluronidase enzyme inhibition were assessed. All results are presented in Table 4.

In the case of antioxidant activity (Figure S3, Supplementary Materials), the percentage of methanol in the extraction mixture and the temperature are statistically significant factors affecting the IC_50_. As expected, in the case of anti-hyaluronidase activity (Figure S4, Supplementary Materials), only the temperature has a statistically significant effect. In that case, effects have a negative sign, that is, as the starting values increase, the IC_50_ decreases.

The antioxidant properties of *P. cuspidati* extract have been widely described in literature data [8]. In the study mentioned above, we indicated that the extract was characterized by the best activity, where 1% *m/w* of β-cyclodextrin was added to the extraction mixture (ethanol: water (5:5 *v:v*)). The antioxidant activity was IC_50_ = 0.16 mg/mL, which is a higher value than the best extract in this study (for W9 IC_50_ = 0.16 mg/mL). Due to the method described in this study, it was possible to obtain a content of resveratrol that is 2 times higher than in the cited study [8]. It is worth noting that both resveratrol and polydatin are characterized by high antioxidant activity [21]; hence, this is shown by the results of the action of the entire extract. For example, Li et al. noted that polydatin was superior to resveratrol in inhibiting malondialdehyde (MDA) production, indicating that polydatin may better protect cells from damage. Therefore, resveratrol and polydatin treatment could be effective for attenuating AAPH-induced oxidative stress in HepG2 cells. Recent results suggest that resveratrol and polydatin were able to alleviate oxidative stress and increase the expression of related antioxidant factors [21]. In the light of these reports, one can explain the higher activity of the W9 extract and the higher content of polydatin. Moreover, it is the justification that the use of extracts shows synergy of active compounds’ action.

In addition to the antioxidant activity of the extracts, there are reports on their anti-inflammatory activity, expressed by inhibiting cyclooxygenase [8,22]. In this study, a different mechanism of anti-inflammatory action was used, i.e., the ability to inhibit the hyaluronidase enzyme. Such activity is shown by the prepared extracts (Table 4). Inhibition of hyaluronidase activity by polyphenolic compounds is related to, among others, the presence of hydroxyl groups. Interestingly, it has been proven that glycones are more potent inhibitors than their corresponding glycosides [23], which indicates the need to monitor the resveratrol content in the prepared extracts.

**Figure 2:**
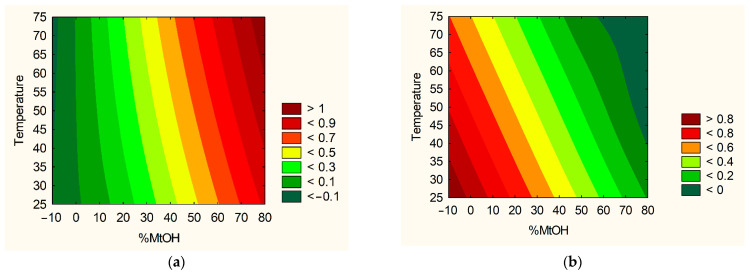
Model utility contour profiles for effect with a positive sign (**a**) and negative sign (**b**) for extract optimalization.

Based on the research results and statistical analyses, it was possible to indicate the technical parameters of the extraction process, leading to an extract with the best properties and the highest activity. The significance of input factors was analyzed, including those with the positive sign (sum of active compounds, TPC) (Figure 2a) and with a negative sign (antioxidant and anti-hyaluronidase activities) (Figure 2b). Based on the utility contour profiles model, it was possible to predict the model and indicate optimized parameters of the extraction process, which are as follows: 70% of methanol in the extraction mixture, temperature 70 °C and 4 cycles (statistically insignificant parameter).

Optimized process parameters were used to prepare the W10 extract, the activity of which was determined to validate the model. The W10 extract was used in the second part of the experimental work, i.e., for the preparation of electrospun nanofibers. Electrospun nanofibers have been formulated to provide a rapidly dissolving pharmaceutical form providing an optimized extract.

Optimized process parameters allowed for the creation of guidelines for the preparation of an extract with the best biological activities (W10 extract). The W10 extract was used in the second part of the experimental work during the preparation of electrospun nanofibers [24]. In this case, the content of extract, PVP and HPβCD was also analyzed (Table 2). A proper solvent mixture was selected to dissolve all compounds and enable nanofibers’ production. The optimization of the electrospinning conditions with respect to the concentration of the polymer, applied voltage, flow rate and distance between the collector and the needle tip was performed, thus producing a continuous stretch of fibers.

The obtained electrospun nanofibers were characterized in terms of their morphology (SEM), structure (XRPD) and possible intermolecular chemical bond formation (FTIR-ATR).

**Figure 3:**
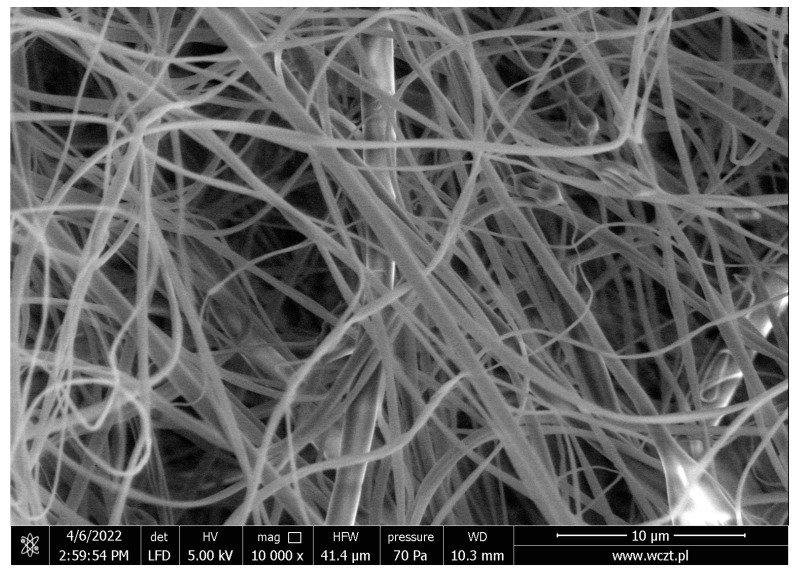
SEM image for nanofibers no. 5.

Scanning electron microscopy for systems no. 2–6 and 9 confirmed the nanofiber structure. The systems (1, 7 and 8) did not create the structure of nanofibers, and it was not possible to remove them from the aluminum foil; therefore, they were not used for further research. The presented SEM image of nanofibers no. 5 (Figure 3) showed that obtained procedure produced the nanostructures with a diameter below 500 nm with homogeneous composition and no defects.

**Figure 4:**
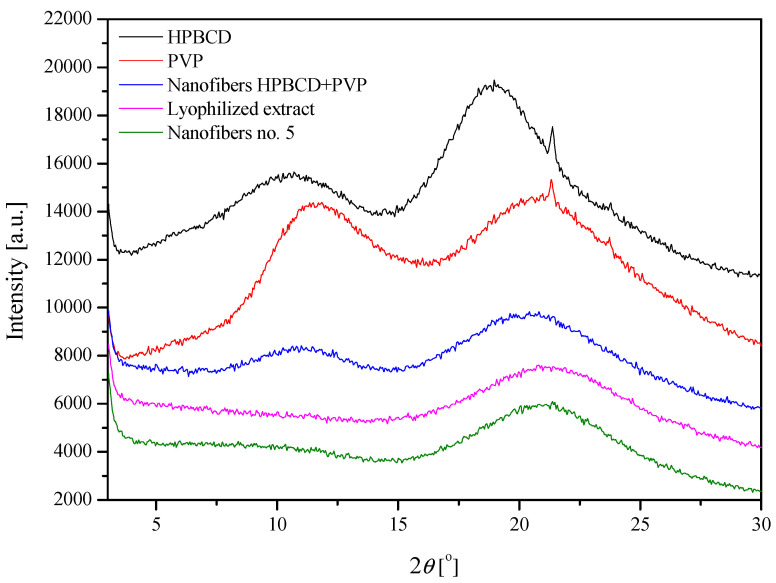
Diffractograms for powder systems and nanofibers no. 5.

The X-ray diffractograms showed that starting materials (lyophilized extract, PVP, HPβCD) display a high broadening of the diffraction peaks, and this with a low intensity indicates the amorphous structure of these systems (Figure 4) [13]. Moreover, background nanofibers produced from PVP and HPβCD, as well as final nanofibers no. 5, are also characterized by amorphousness.

Additionally, it was noted that nanofiber samples spectra show both a polymer matrix and excipient extract presence through the main peak position movement, according to Table 5. The polymer matrix peak position is shifted to a lower 2*θ* angle and above a structural displacement (network expansion), it remains present by the embed excipient and extract. As we may observe for nanofiber HPβCD-PVP vs. nanofiber no. 5 sample comparison, some of polymer matrix micro-strain could be raveled by adding the extract; however, others will grow by the basic structural orientation of the system components. Further analysis for changeable composition and shares of the system may allow for using structural methods for simple predictions of the reaction (a micro-strain shear, network deformation direction and displacement factor) that take place at the nearly amorphous state of the sample.

**Figure 5:**
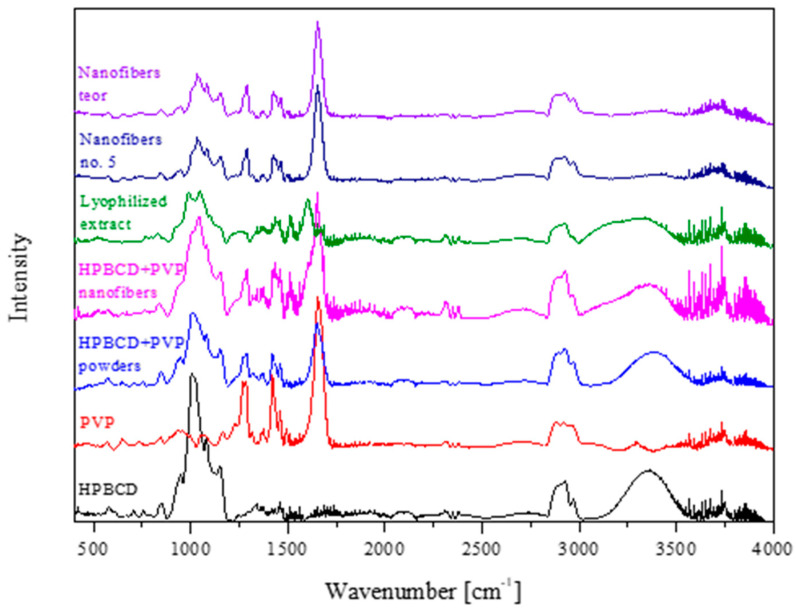
FTIR-ATR spectra for powder samples and nanofiber no. 5.

**Figure 6:**
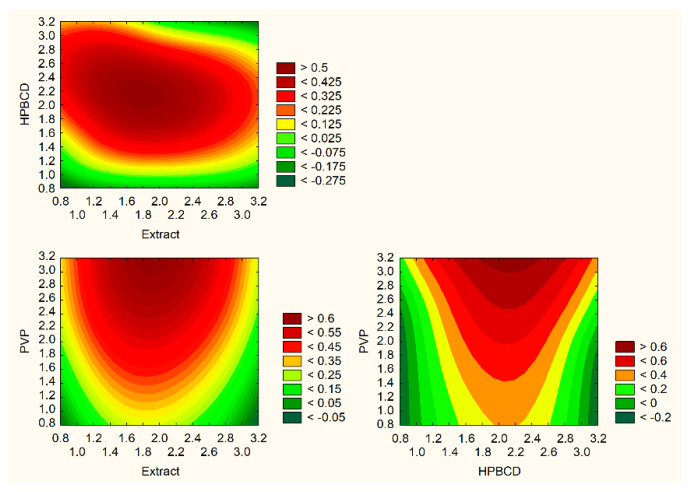
Model utility contour profiles for all effects for electrospun nanofibers optimalization.

At last, the intramolecular interactions between the extract, PVP and HPβCD were analyzed by FTIR-ATR (Figure 5). To determine whether new interactions between the extract and excipients during the electrospinning process occurred, spectra of the lyophilized extract, PVP, HPβCD, background nanofibers produced from PVP and HPβCD, as well as nanofiber no. 5, were taken and presented in Figure 5. Additionally, Figure 6 shows the theoretical sum of the spectra of the lyophilized extract and background nanofibers produced from PVP and HPβCD. Comparing the nanofiber no. 5 and the theoretical spectra, no additional bands or shifting of existing bands were observed, which may indicate no intermolecular interactions between the extract and the excipients.

After confirming that all the analyzed systems have a nanofiber structure, the analysis of the influence of the input parameters (Table 2) on the physicochemical properties of the obtained systems was started.

The first of the analyzed output parameters were the content of active components, which were marked in the prepared nanofibers and are collected in Table 6. While none of the effects was statistically significant (Figure S5, Supplementary Materials), one can notice the trend of the increase in active compounds content when HPβCD content increased in the prepared system. This is related to the possibility of increasing the solubility of the active compound by the greatest water solubility, high amorphization, wetting, solubilizing and complexing of this cyclodextrin [25,26].

**Figure 7:**
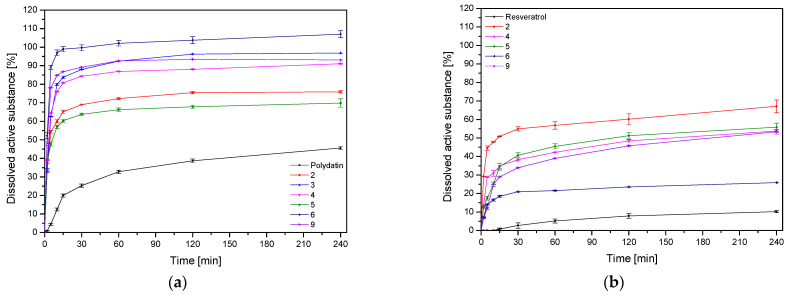
Dissolution profiles of polydatin (**a**) and resveratrol (**b**) from the nanofibers at artificial saliva solution at pH 6.8.

Another critical research was dissolution studies of polydatin and resveratrol from nanofiber (Figure 7, Table 7). There is no guideline for the release method of nanofibers, so in this work, a modified basket method was used. While it is hard to map release medium volume and other physiological conditions located in the oral cavity, the temperature and artificial saliva solution at pH 6.8 closely meet the physiological requirements. It was possible to achieve a 3-fold improvement in the dissolution rate of polydatin, and in the case of resveratrol, there was as much as a 6-fold improvement compared to pure powder compounds. This phenomenon can be explained by the benefits of nanofiber formation, including high load capacity, encapsulation efficiency and a high surface area to volume ratio, all of which can lead to an increase in dissolution rate [13]. PVP-based nanofibers are known to improve the dissolution rate with a burst release of poorly water-soluble compounds [26,27]. This phenomenon can be explained by the following factors: (1) PVP has hygroscopic and hydrophilic properties, and the interactions between polymers and their solvents are more potent than their interactions with one another, so as a result, the polymer chain can quickly absorb solvent molecules, increasing the volume of the polymer matrix and loosening the polymer chains from their rolled shape; (2) a large surface area for the PVP to absorb water molecules increased the porosity for the water molecules to diffuse into the interior of the membrane, and void space for the polymer to swell and disentangle and for the dissolved substance molecules to disperse into the bulk dissolution medium can all be provided by the membrane’s three-dimensional continuous web structure; (3) composites were created between the drug and the matrix polymer at the molecular level, allowing for the dissolution of both PVP and substance molecules [28].

The total amount of released polydatin and resveratrol from nanofibers at 15 min was assessed to determine the significance of the input factors. Again, while none of the effects was statistically significant (Figure S6, Supplementary Materials), it can be seen that the increase in extract and HPβCD content in the prepared system was influenced by the rise in the content of released substances. This is again related to the solubilizing properties of the cyclodextrin used in systems, so we can see how crucial the presence of HPβCD is in the prepared nanofibers.

**Figure 8:**
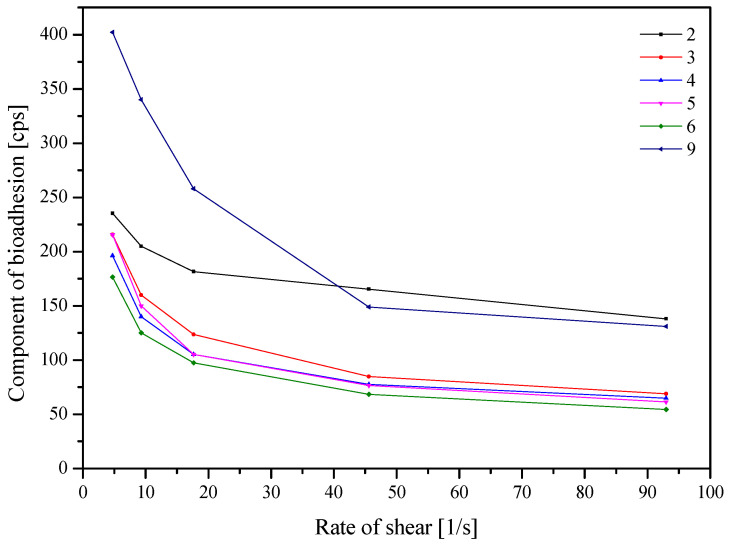
Component of bioadhesion of the nanofibers.

An important factor determining the application properties of a buccal product is the ability to bind the polymer to the mucin located in the mucosa. For this purpose, rheological studies of nanofiber mixtures were used, and the results are presented in the Figure 8.

When analyzing the significance of the input factors (Figure S7, Supplementary Materials), it can be noticed that the increase in bioadhesion is noticeable with the increase in the amount of PVP and HPβCD in the samples. PVP is a polymer with good biodegradability, biocompatibility and low toxicity, commonly used as a matrix for controlled drug delivery. Its mucoadhesiveness is explained by the interaction of carbonyl groups with mucin through the presence of hydrogen bonds and Van der Walls forces [29,30]. While cyclodextrins are not typical mucoadhesive compounds, their mucoadhesive properties are notable, perhaps due to the molecules’ hydrophilic outer part, which can form hydrogen bonds with hydroxyl groups on sugars and other O- and *N*-containing groups on the mucosal protein backbone [31].

As previously mentioned, the oral cavity becomes an exciting site for administering polyphenols, especially those undergoing rapid first-pass metabolism, which results in low bioavailability, as in the case of resveratrol [32]. For this purpose, apparent permeability coefficients for standards and active compounds from extract W10 and nanofibers were assessed (Table 8).

While none of the effects was statistically significant (Figure S8, Supplementary Materials), it can be seen that the content of the extract and HPβCD was the factor most influencing the permeation coefficients. Again, HPβCD improves the solubility of active compounds, and those in the dissolved form are more available for transport across the membrane by passive diffusion. Therefore, the use of cyclodextrins is one of the approaches to increase the penetration of resveratrol through biological membranes by the solubility improvement and permeability enhancement through the unstirred water layer [25,33]. There are also reports of a significant influence of the nanofibers structure on the resveratrol penetration through the skin, specifically through the stratum corneum and into the epidermal and dermal layers [13]. This is worth noting, as the increased content of PVP in the system limited permeation through the membrane, which is associated with increased system viscosity. Permeability is inversely proportional to the viscosity of the fluid, which confirms the obtained results.

Dissolution of a drug in an aqueous environment is almost always a prerequisite for oral absorption, and thus, insufficient water solubility often results in limited oral bioavailability. Intestinal permeability is, in addition to water solubility, a key parameter that governs oral absorption [34]. Thus, solubility and permeability are key factors affecting oral bioavailability [35]. Therefore, it is possible to extrapolate the obtained results and suggest the possibility of increasing the bioavailability with the use of buccal electrospun nanofibers together with the increased solubility and permeability of active compounds.

Finally, to confirm the maintenance of the antioxidant properties of the extract, such studies have also been carried out for nanofibers by using the DPPH method. The activity of W10 was assessed and amounted to IC_50_ = 0.13 ± 0.01 mg/mL. The IC_50_ for nanofibers in each case remained within the error limits obtained for the W10 extract. Therefore, it confirmed that the electrospinning process does not affect the biological properties of the processed extract.

To summarize the production of electrospun nanofibers step, a model of utility contours of profiles was made for all the tested effects (Figure 6). Based on the analysis of the Figure 5, the best composition of electrospun nanofibers was selected, and it is the ratio of extract/PVP/HPβCD 1:1:1 *w/w/w*, i.e., the design of nanofiber number 5.

## 4. Conclusions

The use of the Design of Experiments (DoE) in the extraction of plant material will be essential in advancing and modernizing the creation of standardized phytotherapy. In our study, the influence of the parameters (composition of the extraction mixture, its temperature and number of extraction cycles) on biological properties of the obtained extracts was assessed. It was shown that 70% of methanol in the extraction mixture, temperature 70 °C and 4 cycles are the optimal parameters for extraction of active compounds, such as resveratrol and polydatin from the *P. cuspidati* rhizome and root.

The DoE approach can also be successfully used to select the optimal electrospinning parameters. In the present study, PVP/HPβCD-loaded blend and core shell nanofibers with smooth and bead-less morphology were successfully fabricated for application as new and controlled drug delivery extract systems. The release rate of active compounds in electrospun nanofibers increased with the increase of HPβCD ratio due to enhanced hydrophilicity of the nanofibers. The best composition of electrospun nanofibers was selected, and it is the ratio of extract/PVP/HPβCD 1:1:1 *w/w/w*.

Thus, the PVP/HPβCD-based electrospun nanofibers might be strong enough to be easily inserted within the oral cavity and immediate release the incorporated bioactives, while ensuring patient compliance with the smooth structure of nanofibers and low stiffness during treatment. These characteristics, along with the proven antioxidant and anti-inflammatory properties of *P. cuspidati* extract, suggest the particular benefits of the buccal delivery system as a promising strategy to improve the bioavailability of bioactives.

## Figures and Tables

**Table 1:** Factorial Extraction Process Experiment Plan.

No.	% of Methanol in the Extraction Mixture	Temperature	Number of Cycles
W1	0	30	3
W2	0	50	5
W3	0	70	4
W4	35	30	5
W5	35	50	4
W6	35	70	3
W7	70	30	4
W8	70	50	3
W9	70	70	5

**Table 2:** Factorial Electrospinning Process Experiment Plan.

No.	Content		
	W10 Extract (g)	HPβCD (g)	PVP (g)
F1	1	1	1
F2	1	2	3
F3	1	3	2
F4	2	1	3
F5	2	2	2
F6	2	3	1
F7	3	1	2
F8	3	2	1
F9	3	3	3

**Table 3:** Content of Active Compounds in the Extracts.

No.	Content (µg/1 g Plant Material) Mean ± SD				Sum of Active Compounds (µg/1 g Plant Material) Mean	TPC (mg GAE/1 g Plant Material) Mean ± SD
	Polydatin	Resveratrol	Emodin	Parietin		
W1	174.29 ± 0.68	185.85 ± 10.75	7.85 ± 0.41	0.08 ± 0.02	452.61	7.85 ± 0.41
W2	288.76 ± 22.64	206.69 ± 3.99	11.97 ± 0.62	0.23 ± 0.02	729.16	11.97 ± 0.62
W3	1384.68 ± 6.51	307.38 ± 1.57	12.95 ± 1.95	0.18 ± 0.01	1892.28	12.95 ± 1.95
W4	1060.43 ± 8.74	324.95 ± 15.49	20.35 ± 0.72	0.19 ± 0.08	1631.68	20.35 ± 0.72
W5	1388.60 ± 66.33	353.33 ± 12.53	21.96 ± 0.88	0.20 ± 0.01	2001.47	21.96 ± 0.88
W6	2891.96 ± 26.31	369.26 ± 3.88	23.11 ± 1.06	0.15 ± 0.02	3738.75	23.11 ± 1.06
W7	2730.72 ± 30.61	414.12 ± 5.30	23.09 ± 2.22	0.29 ± 0.02	3931.77	23.09 ± 2.22
W8	4179.04 ± 29.44	449.50 ± 1.24	30.75 ± 2.14	0.13 ± 0.08	4925.62	30.75 ± 2.14
W9	4199.43 ± 68.10	652.67 ± 17.44	35.01 ± 1.77	0.14 ± 0.02	5241.30	35.01 ± 1.77

**Table 4:** Antioxidant and Anti-Hialuronidase Activities.

No.	DPPH IC_50_ (mg/mL)	ABTS IC_50_ (mg/mL)	CUPRAC IC_50_ (mg/mL)	FRAP IC_50_ (mg/mL)	Hyaluronidase Inhibition IC_50_ (mg/mL)
Mean ± SD					
W1	0.80 ± 0.02	0.66 ± 0.08	2.16 ± 0.02	0.49 ± 0.03	73.52 ± 2.37
W2	0.65 ± 0.01	0.49 ± 0.04	1.63 ± 0.03	0.30 ± 0.01	44.02 ± 1.48
W3	0.50 ± 0.01	0.42 ± 0.04	1.33 ± 0.30	0.29 ± 0.01	33.21 ± 2.40
W4	0.45 ± 0.01	0.44 ± 0.04	0.45 ± 0.02	0.23 ± 0.01	25.45 ± 0.87
W5	0.31 ± 0.02	0.38 ± 0.03	0.30 ± 0.02	0.18 ± 0.01	19.98 ± 1.67
W6	0.25 ± 0.01	0.31 ± 0.02	0.27 ± 0.03	0.13 ± 0.01	11.34 ± 2.45
W7	0.22 ± 0.03	0.21 ± 0.04	0.19 ± 0.01	0.14 ± 0.01	10.43 ± 1.33
W8	0.16 ± 0.02	0.18 ± 0.02	0.13 ± 0.01	0.11 ± 0.01	4.69 ± 0.33
W9	0.13 ± 0.01	0.14 ± 0.01	0.11 ± 0.01	0.09 ± 0.01	4.35 ± 0.28
	IC_50_ (µg/mL)	IC_50_ (µg/mL)	IC_50_ (µg/mL)	IC_50_ (µg/mL)	
Resveratrol	22.32 ± 0.20	10.84 ± 0.23	20.82 ± 1.29	9.17 ± 0.68	
Polydatin	35.06 ± 1.11	25.21 ± 2.32	68.73 ± 0.48	12.90 ± 0.29	

**Table 5:** XRPD Signals’ Positions.

Sample	PVP	HPβCD	Lyophilized Extract	Nanofiber HPBCD-PVP	Nanofiber no. 5
(1) Peak position [2*θ*]	11.45	10.26	-	11.11	9.48
(2) Peak position [2*θ*]	21.28	18.72	21.37	20.61	21.16
Matrix peak position displacement [2*θ*]	-	-	-	(1) −0.34 (2) −0.67	(1) −1.97 (2) −0.12
Matrix peak position displacement [Å]			-	(1) 0.24 (2) 0.13	(1) 1.76 (2) 0.02

**Table 6:** Content of Active Components in Nanofibers.

	Nanofibers 2	Nanofibers 3	Nanofibers 4	Nanofibers 5	Nanofibers 6	Nanofibers 9
	Content (µg/100 mg Nanofibers)					
Solvent: methanol						
Polydatin	5.16 ± 0.11	8.35 ± 0.11	13.66 ± 0.87	33.32 ± 1.60	15.04 ± 3.48	9.45 ± 0.33
Resveratrol	3.70 ± 0.01	1.28 ± 0.01	5.93 ± 0.59	8.60 ± 0.39	1.68 ± 0.33	1.05 ± 0.04
Solvent: artificial saliva solution at pH 6.8						
Polydatin	8.20 ± 0.01	6.96 ± 0.06	15.46 ± 0.01	27.46 ± 1.08	18.62 ± 0.27	19.24 ± 0.08
Resveratrol	0.81 ± 0.03	0.60 ± 0.03	1.02 ± 0.01	11.00 ± 0.52	1.79 ± 0.02	2.26 ± 0.05

**Table 7:** Total Amount of Released Polydatin and Resveratrol from Nanofibers at 15 Minutes.

	Nanofibers 2	Nanofibers 3	Nanofibers 4	Nanofibers 5	Nanofibers 6	Nanofibers 9
	Total Amount of Released Drug from 100 mg of Nanofibers (µg) at 15 min					
Polydatin	5.75 ± 0.07	5.83 ± 0.04	12.50 ± 0.03	16.56 ± 0.22	18.80 ± 0.29	17.47 ± 0.02
Resveratrol	0.31 ± 0.01	0	0.31 ± 0.02	3.80 ± 0.20	0.26 ± 0.01	0.56 ± 0.01

**Table 8:** Apparent Permeability Coefficients for Standards, as well as Active Compounds from Extract W10 and Nanofibers.

	Standards	W10	Nanofibers 2	Nanofibers 3	Nanofibers 4	Nanofibers 5	Nanofibers 6	Nanofibers 9
	Apparent Permeability Coefficient P_app_ × 10^−6^ (cm/s)							
Polydatin	0.0036 ± 0.0001	0.0096 ± 0.0007	3.6213 ± 0.4921	0.7235 ± 0.0460	0.1740 ± 0.0043	0.2060 ± 0.0155	0.0455 ± 0.0039	0.0088 ± 0.0003
Resveratrol	1.0924 ± 0.0778	0.0281 ± 0.0017	47.5107 ± 6.1468	11.7056 ± 0.6146	11.3306 ± 0.2093	13.4603 ± 0.9008	0.1387 ± 0.0070	0.1102 ± 0.0042

## Data Availability

Not applicable.

## Supplementary Materials

The following supporting information can be downloaded at: https://www.mdpi.com/article/10.3390/nu14193897/s1, Table S1: Validation parameters; Figure S1: Pareto plot of standardized effects for the sum of active compounds; Figure S2: Pareto plot of standardized effects for the TPC; Figure S3: Pareto plot of standardized effects for the antioxidant activity using DPPH assay; Figure S4: Pareto plot of standardized effects for the anti-hyaluronidase activity; Figure S5: Pareto plot of standardized effects for content of polydatin (a) and resveratrol (b) dissolved in methanol, and polydatin (c) and resveratrol (d) dissolved in artificial saliva solution at pH 6.8; Figure S6: Pareto plot of standardized effects for the total amount of released polydatin (a) and resveratrol (b) from nanofibers at 15 min; Figure S7: Pareto plot of standardized effects for component of bioadhesion; Figure S8: Pareto plot of standardized effects for the apparent permeability coefficients of polydatin (a) and resveratrol (b).
