# Supplementary material for: Is It Possible to Improve the Bioavailability of Resveratrol and Polydatin Derived from Polygoni cuspidati Radix as a Result of Preparing Electrospun Nanofibers Based on Polyvinylpyrrolidone/Cyclodextrin?
Source: Nutrients. 2022 Sep 21;14(19):3897. doi: 10.3390/nu14193897 (PMC9572329; doi:10.3390/nu14193897)
Supplement: Supplementary file 1 [file nutrients-14-03897-s001.zip › nutrients-1914286-supplementary.pdf]

Improving the bioavailability of resveratrol and polydatin derived from *Polygoni cuspidati* radix as a result of preparing electrospun nanofibers based on polyvinylpyrrolidone/cyclodextrin

# - Supplementary material

Table S1. Validation parameters

Parameter	Polydatin	Resveratrol	Emodin	Parietin
Linearity: y = ax + b				
$a \pm S_a$	0.276 ± 0.008	0.607 ± 0.011	0.114 ± 0.002	31.366 ± 0.205
$b \pm S_b$	insignificant (α=0.05)	insignificant (α=0.05)	insignificant (α=0.05)	insignificant (α=0.05)
Correlation coefficient (r)	0.999	0.999	0.999	0.999
Range of linearity [µg/mL]	144.0–1440.0	27.0–270.0	4.0 – 40.0	0.01 – 0.1
Intra-day precision, RSD (<5% required) = repeatability				
The lowest	0.425	0.038	3.055	2.912
The middle	0.288	0.237	0.428	3.454
The lowest	0.378	0.099	2.534	0.603
Limit of detection (LOD) [µg/mL]	91.53	11.42	1.99	0.01
Limit of quantification (LOQ) [µg/mL]	277.36	34.60	6.05	0.04

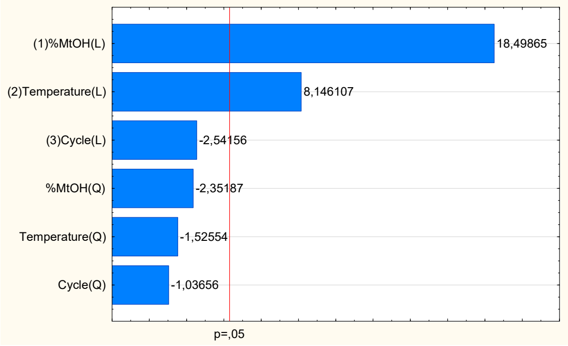

Figure S1. Pareto plot of standardized effects for the sum of active compounds.

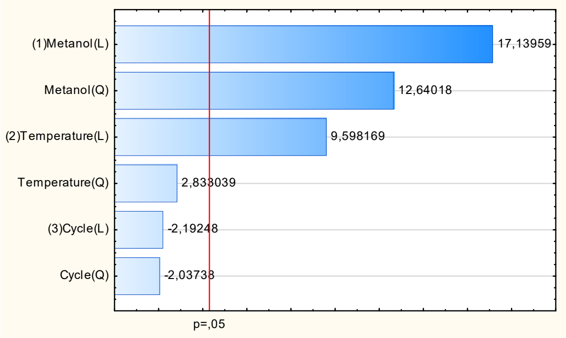

Figure S2. Pareto plot of standardized effects for the TPC.

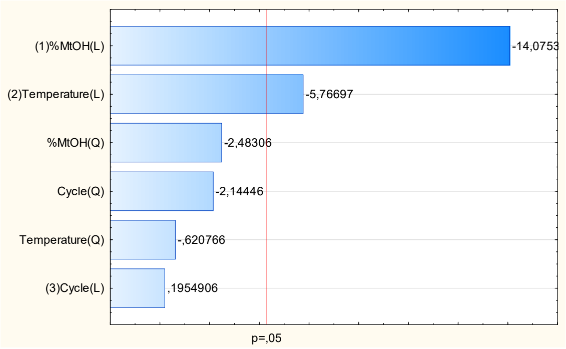

Figure S3. Pareto plot of standardized effects for the antioxidant activity using DPPH assay.

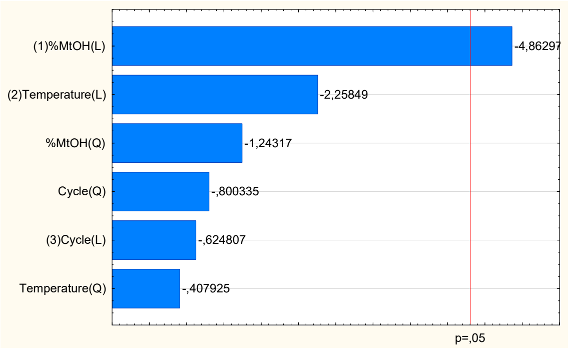

Figure S4. Pareto plot of standardized effects for the anti-hyaluronidase activity.

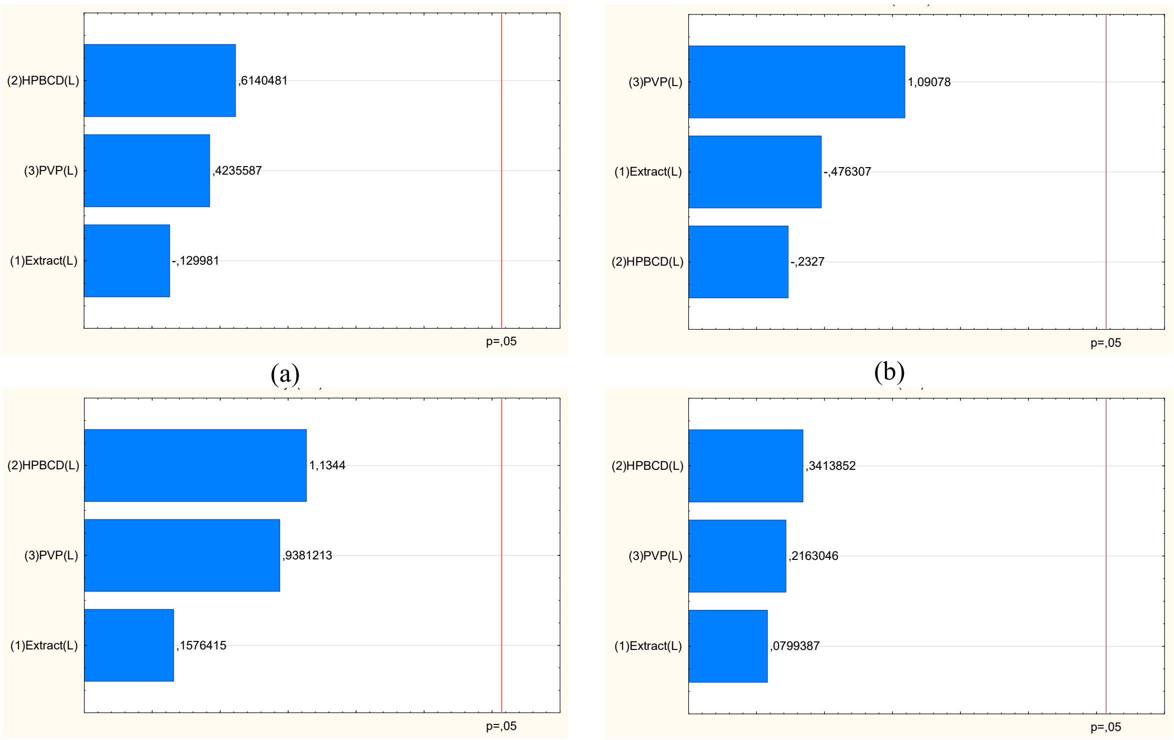

(c) (d)

Figure S5. Pareto plot of standardized effects for content of polydatin (a) and resveratrol (b) dissolved in methanol, and polydatin (c) and resveratrol (d) dissolved in artificial saliva solution at pH 6.8.

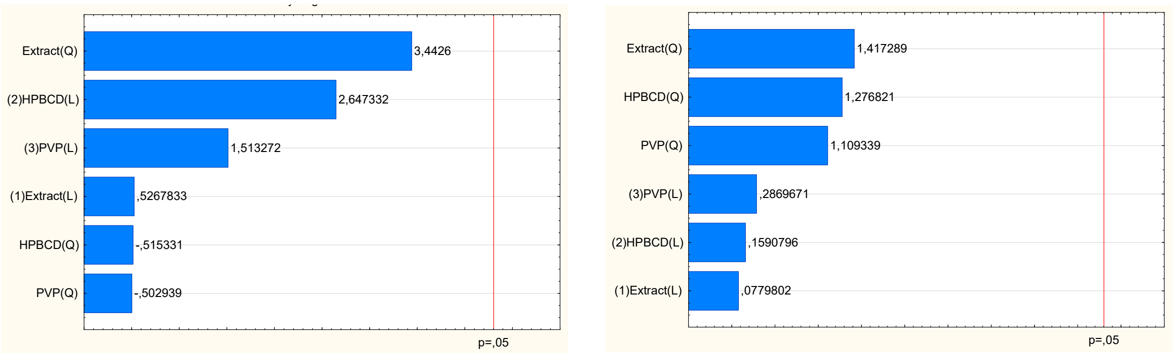

(a) (b)

Figure S6. Pareto plot of standardized effects for the total amount of released polydatin (a) and resveratrol (b) from nanofibers at 15 minutes.

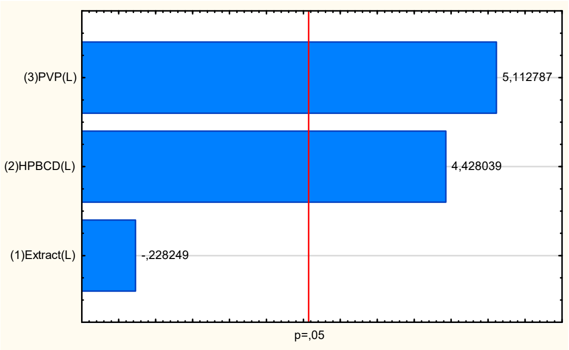

Figure S7. Pareto plot of standardized effects for component of bioadhesion.

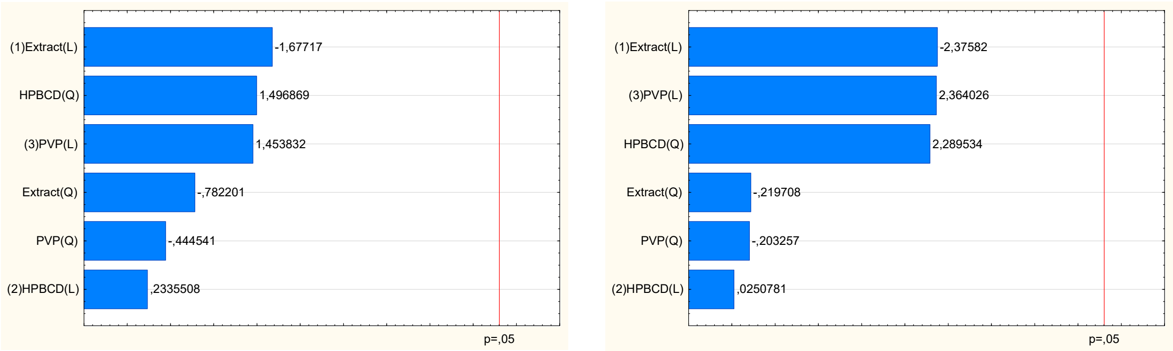

(a) (b)

Figure S8. Pareto plot of standardized effects for the apparent permeability coefficients of polydatin (a) and resveratrol (b).
